# Cytomegalovirus-Immune Recovery Retinitis After Initiation of Highly Active Antiretroviral Therapy: A Case Series

**DOI:** 10.3389/fmed.2022.807013

**Published:** 2022-04-27

**Authors:** Yiwen Qian, Luoziyi Wang, Jing Jiang, Jinshan Suo, Huan Weng, Xin Che, Hongzhou Lu, Zhiliang Wang

**Affiliations:** ^1^Department of Ophthalmology, Huashan Hospital of Fudan University, Shanghai, China; ^2^Department of Infectious Disease, Shenzhen Third People's Hospital, Shenzhen, China

**Keywords:** cytomegalovirus retinitis, human immunodeficiency virus, highly active antiretroviral therapy, cytomegalovirus-immune recovery retinitis, case series

## Abstract

**Purpose:**

To delineate the characteristics and treatment of cytomegalovirus-immune recovery retinitis (CMV-IRR) in human immunodeficiency virus (HIV) patients with immune recovery under effective highly active antiretroviral therapy (HAART) regimen.

**Methods:**

We reported four patients with HIV who were diagnosed with CMV-IRR soon after effective HAART. Plasma levels of CD4 T cells, HAART regimen, and other clinical and laboratory characteristics of the four patients were described. Patients were monitored for ocular manifestations and clinical signs under effective ocular and systemic anti-cytomegalovirus (CMV) and corticosteroid treatment for 12 months.

**Results:**

With HAART, plasma levels of CD4 T cell counts rose remarkably. The mean baseline CD4 count of the four patients was 14.5 (range from 7 to 33) cells/μl before HAART and 183.25 (range from 153 to 220) cells/μl when diagnosed with CMV-IRR. Ophthalmic examination demonstrated severe vitreous opacities and necrotizing retinitis, intraretinal hemorrhages, and vasculitis. A large number of CMV sequencing was detected by DNA sequencing of vitreous samples. All four patients were recovered from CMV-IRR with anti-CMV and corticosteroid treatment.

**Conclusions:**

Cytomegalovirus-immune recovery retinitis is a new diagnosis of HIV-associated ocular complication under HAART. These findings suggest that the immunological effects of HAART may accelerate the CMV retinitis in patients with very low initial CD4 T cell counts. HIV patients are recommended to have a thorough fundus examination before HAART initiation and a close follow-up especially in those with low CD4 counts to avoid the progression of CMV retinitis.

## Introduction

In the pre-highly active antiretroviral therapy (HAART) era, cytomegalovirus retinitis (CMVR) is the most common intraocular opportunistic infection in patients with Acquired Immune Deficiency Syndrome (AIDS) and occurs primarily in patients with an absolute CD4 counts <50 cells/μl. HAART was introduced in 1996 to treat HIV-infected patients by reducing HIV viral load and increasing CD4 T cell counts. The most commonly used combination of HAART consists of one protease inhibitor and two reverse transcriptase inhibitors, resulting in increased life survival and a decrease in the incidence of CMVR.

Over the past 20 years, the HAART-mediated improvement of immune function in patients with AIDS may also alter the way in which the eye responds to cytomegalovirus (CMV), resulting in a change in the clinical manifestations of ocular CMV retinitis.

Immune reconstitution inflammatory syndromes (IRIS) represent an inflammatory response to an opportunistic pathogen in the context of immune recovery after initiating HAART (1). Immune recovery uveitis (IRU) is characterized by anterior segment and vitreous inflammatory reactions that are directed toward CMV antigens in ocular tissues, accompanied by cystoid macular edema (CME) and epiretinal membrane formation (2).

Immune recovery uveitis occurs in some patients with pre-existing CMVR due to the improved immune function associated with new potent antiretroviral. It has been described in the context of inactive retinitis several months to years after HAART initiation (3). In contrast, active CMV retinitis in immune reconstitution subjects had only been documented in a few case series with complex and severe retinal lesions and was postulated as an IRIS phenomenon, called “immune recovery retinitis” (4).

Therefore, in this retrospective study, we reported four patients with active CMV retinitis and severe vitritis in the context of a successful HAART regimen. They all had very low CD4 T cells before HAART initiation and were then developed to CMV-IRR within 8 weeks in good response to HAART. We depicted the clinical signs and the DNA sequencing of the vitreous body and followed up with them for 12 months with effective treatment.

## Methods

### Patients and Methods

We reviewed four patients who attended our department and were diagnosed with active CMV-IRR with high CD4 counts under effective HAART from May 2019 to January 2021. We retrospectively studied the medical records of the four patients including clinical manifestations, ophthalmic examinations, and treatments. The study was approved by the institutional ethics committee of Huashan Hospital affiliated with Fudan University (protocol number: KY2021-837), and the treatment was performed under the tenets of the Declaration of Helsinki. The patients enrolled in the study signed the written informed consent for the publication of their data and examinations. Demographic data, CD4 cell counts, and current HAART regimen were all recorded. The CD4 counts were done using flow cytometry in all four patients. Ophthalmological examinations were performed using a slit lamp, fundoscopic investigation, fundus photographs, ocular B-ultrasound, and optic coherent tomography (OCT) (ZEISS, CIRRUS HD-OCT 4000, Germany).

### Signs and Symptoms of CMV-IRR

Diagnosis of IRIS was specified as follows: (1) good response to ART; (2) deterioration of an infectious condition related to ART initiation; and (3) inability to explain the symptoms.

Cytomegalovirus retinitis was defined by necrotizing retinitis, intraretinal hemorrhages, and vasculitis (manifested as yellow-white retinal lesions with granular border and hemorrhage along with vessels). Retinitis improvement was defined as the replacement of hemorrhages with atrophic scar (5). The location of the CMVR lesion was categorized into three zones. Zone 1 consisted of the area within 1,500 μm of the edge of the optic nerve or within 3,000 μm of the center of the fovea. Zone 2 was extended from the limits of zone 1 to a circle defined by the ampullae of the vortex veins. Zone 3 was extended from the limits of zone 2 to the ora serrata.

Patients with CMV-IRR were defined as those who developed CMV retinitis or worsening CMV retinitis with vitritis on successful HAART (4). The CMV-IRR was diagnosed by an experienced ophthalmologist by fundoscopic exploration and vitreous sample DNA sequencing (BGI, China).

Immune recovery uveitis was defined by completely healed CMV retinitis with any of the following types of ocular inflammation under successful ART: anterior uveitis, vitritis, papillitis, cystoid macular edema, or epiretinal membrane (6).

Moreover, HIV retinopathy, toxoplasmosis retinitis, acute retinal necrosis, progressive outer retinal necrosis, and syphilitic retinochoroiditis were excluded by laboratory assessment and fundus manifestations.

### Treatment of CMV-IRR

All four patients underwent three-port pars plana vitrectomy (PPV) for vitreous samples DNA sequencing with or without silicone oil tamponades. They all received appropriate anti-CMV therapy during the follow-up that includes oral ganciclovir (2 g/tid) and intravitreal injections. Intravitreal treatment regimen included ganciclovir (2 mg/0.1 ml) and dexamethasone (0.4 mg) injections (once a week for the first months and transferred once every other week until the lesion border was stable). All four patients received oral ganciclovir treatment for 6 months and the mean intravitreal injection was 7.25 times for an eye (range from 6 to 10).

## Results

**Figure 1:**
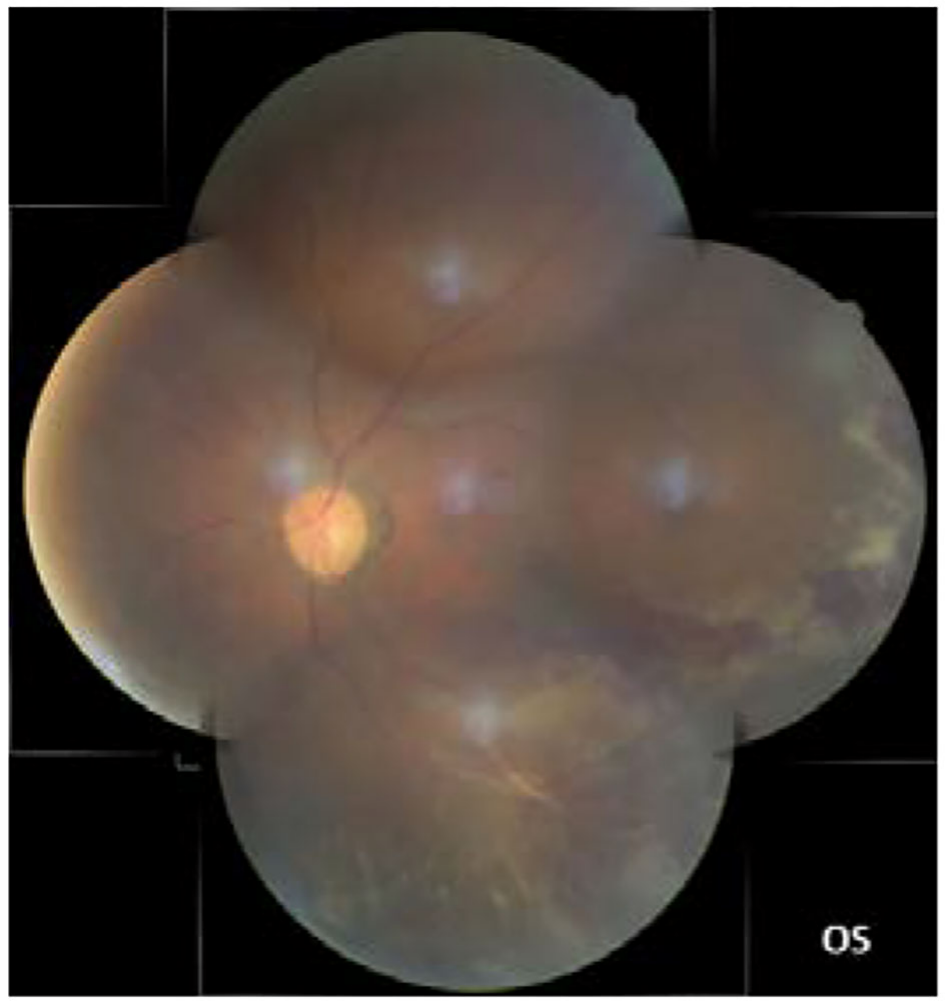
The fundus showed typical necrotizing retinitis, intraretinal hemorrhages, and vasculitis in zones 1–3 (the left eye of patient four).

**Figure 2:**
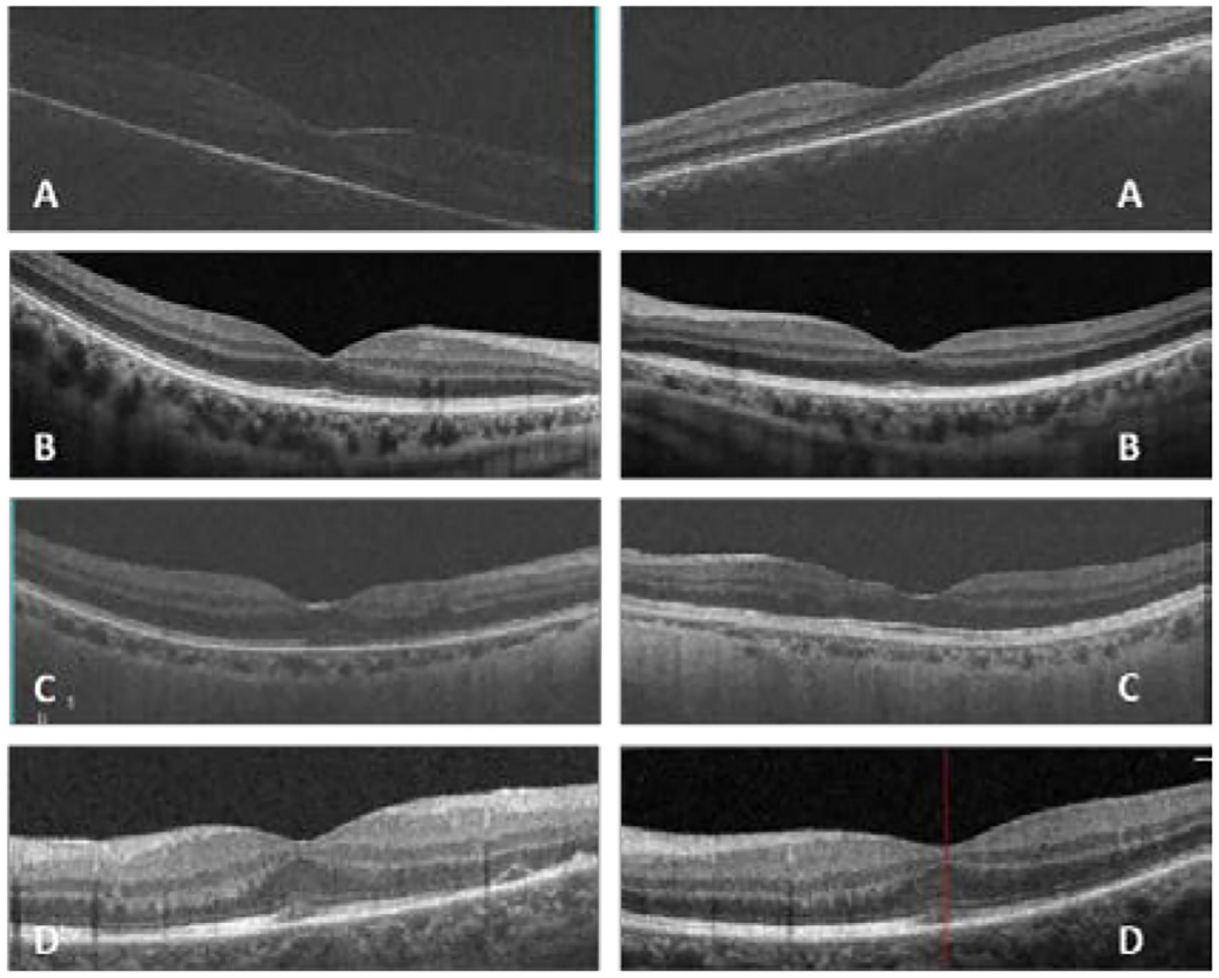
In patient one **(A)**, optic coherent tomography (OCT) showed an epiretinal membrane in macular in the right eye; in patient two **(B)**, OCT was normal in macular in both eyes; in patient three **(C)**, OCT showed a slight epiretinal membrane and inner retina disorder in macular in both eyes; in patient four **(D)**, OCT showed a slight epiretinal membrane and a minor retinal neurosensory layer detachment in the left eye (Left: the right eye, right: the left eye, **(D)** shows only the left eye for the atrophy of the right eye).

**Figure 3:**
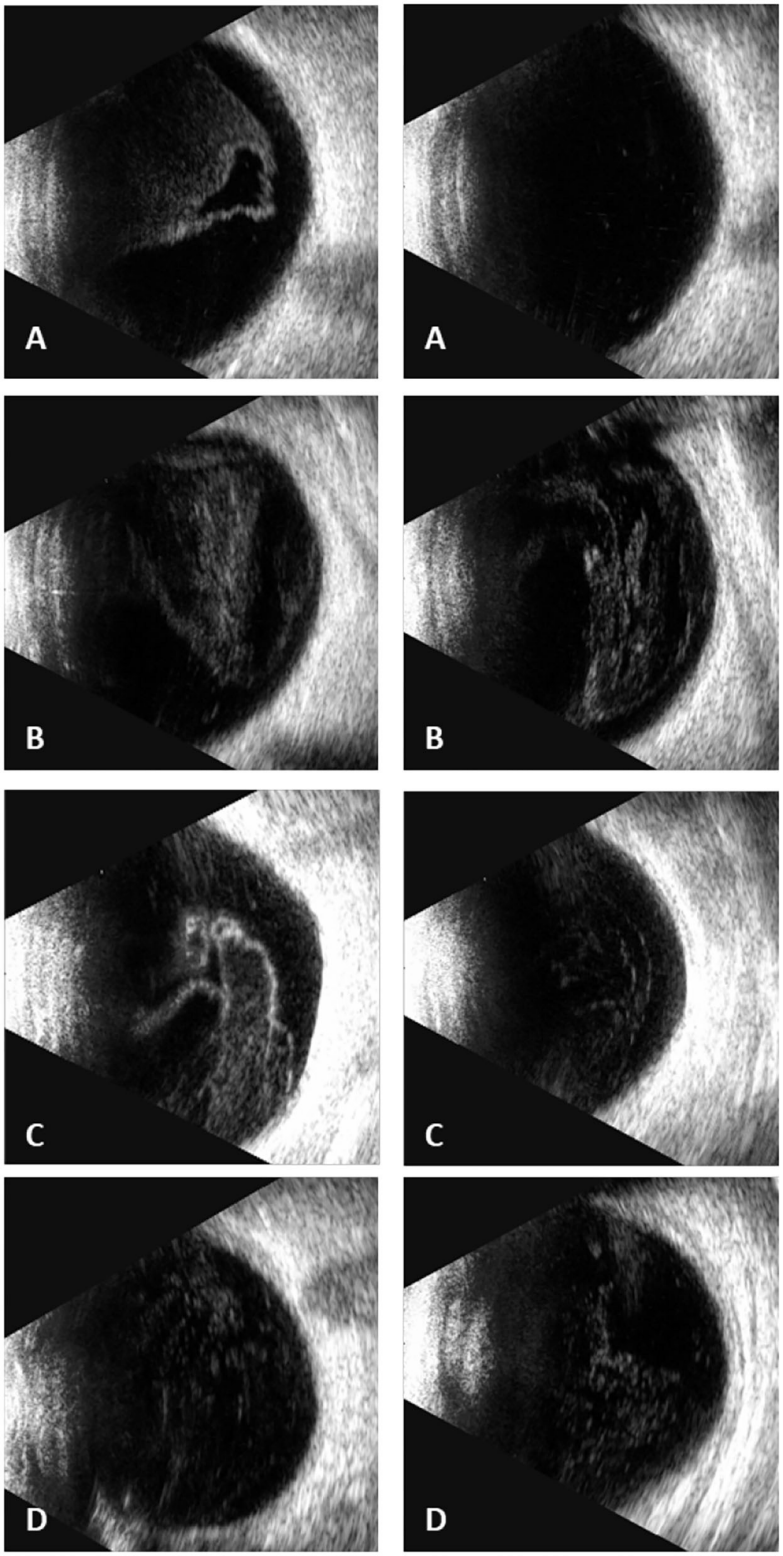
Ultrasound B scans demonstrated severe vitritis in all four patients [**(A)** patient one, **(B)** patient two, **(C)** patient three, **(D)** patient four] (Left: the right eye; right: the left eye) [**(D)** shows only the left eye for the atrophy of the right eye].

In our study, all four patients were progressed with active CMVR lesions with immune recovery within 8 weeks of HAART initiation. Characteristics of the four CMV-IRR patients with HIV positive are summarized in Table 1. Among the four patients, the mean CD4 T cells were 14.5/μl at the HIV diagnosis and 183.25/μl at the CMV-IRR diagnosis. All four patients had an obvious increase of CD4 T cells over 100/μl from a very low baseline (<40/μl). They came to our ophthalmology department and presented with progressed blurred vision without any ophthalmological examination or treatment. The slit lamp showed vitritis and retinitis without anterior segment abnormalities. The vitreous activity was evaluated according to the grading system (1+ to 4+) proposed by Nussenblatt et al. (7). Fundus examination including mydriasis fundus examinations, ultrasound B scans, and OCT scans was performed in the four patients. The fundus showed typical necrotizing retinitis, intraretinal hemorrhages, and vasculitis (Figure 1, patient four). Three patients had retina lesions in zones 2 and 3, while the other patient had retina involvement in zones 1–3. OCT showed normal in one patient and a slight epiretinal membrane in macular in two patients. The other patient had a minor retinal neurosensory layer detachment in the macular (Figures 2A–D). Ultrasound B scans demonstrated obvious vitritis in all four patients (Figures 3A–D). PPV was done and vitreous samples were aspirated for next-generation sequencing. The results demonstrated a high copy of the CMV sequence ranging from 60,527 to 445,532.

**Table 1:** Characteristics of the four cytomegalovirus-immune recovery retinitis (CMV-IRR) patients with human immunodeficiency virus (HIV) positive.

	**Patient**			
	**1**	**2**	**3**	**4**
Age	54	40	50	40
Gender	Male	Male	Male	Male
AIDS diagnosis				
Eye with CMV-IRR	Right	Both	Both	Left
CD4 counts/μl				
Baseline	10	8	33	7
CVMR diagnosis	200	160	220	153
HAART				
Drug Week	• AZT,3TC,EFV • 8	• 3TC,DTG • 7	• 3TC,EFV,TDF • 4	3TC,EFV,TDF 8
Visual acuity	• R 20/166 • L 20/25	• R 20/50 • L 20/40	• R 20/25 • L 20/50	R NLP L 20/66
IOP	R15 L16	R20 L17	R8 L8	L14
• Vitreous • CVMR region	• +++ • Zone 2,3	• ++ • Zone 2,3	• ++ • Zone 2,3	++ Zone 1,2,3
Surgery	PPV+silicone oil tamponades	PPV+silicone oil tamponades	PPV	PPV
Intravitreal injection				
Drugs Times	• Ganciclovir+TA • 10	• Ganciclovir+TA • 8	• Ganciclovir+TA • 6	Ganciclovir+TA 6
• Surgery complications • CMV copies	• NO • 66,544	• Retinal detachment • 446,072	• No • 60,527	Vitreous hemorrhage 206,679
• Outcomes after • 12 months	• R 20/100 • L 20/25	• R 20/20 • L 20/20	• R 20/25 • L 20/40	R NLP L 20/50

*3TC, Lamivudine; DTG, Dolutegravir; EFV, Efavirenz; TDF, Tenofovir; TA, dexamethasone; NLP, no light perception*.

Then, the patients underwent weekly intravitreal injection of ganciclovir 2 mg/0.1 ml plus steroids/dexamethasone 0.4 mg for the first month and shifted to biweekly till the lesion was stable. Oral use of ganciclovir was administered as 2 g tid for 6 months.

### Patient 1

A 54-year-old male experienced blurred vision in the right eye 2 months after HAART initiation (Zidovudine [AZT], Lamivudine [3TC], and Efavirenz [EFV]). CD4 counts were 200/μl. Visual Acuity (VA) was 20/166 in the right eye. Ophthalmic examination showed retinal necrosis and hemorrhages with severe vitritis (+++) in zones 2 and 3. OCT showed a slight epiretinal membrane in the macular of the right eye. He underwent PPV and silicone oil tamponades, and silicone oil removal was conducted 6 months later. Vitreous DNA sequencing showed CMV sequence of 66,544 copies. A total of 10 intravitreal injections were performed until the lesions were stable. His VA of the right eye was 20/100 at the last follow-up.

### Patient 2

A 40-year-old male experienced blurred vision and black shadows in both eyes 2 months after HAART initiation (3TC and Dolutegravir [DTG]). CD4 counts were 160/μl. VA was 20/50 in the right eye and 20/40 in the left eye. Ophthalmic examination showed retinal necrosis and hemorrhages with vitritis (++) in zones 2 and 3. OCT was normal in the macular. He underwent PPV and silicone oil tamponades in the left eye and ganciclovir and steroid intravitreal injections for both eyes. Afterward, immediate PPV and silicone oil tamponades were conducted in the opposite eye for secondary retinal detachment 1 month later. Vitreous DNA sequencing showed a CMV sequence of 446,072 copies. Silicone oil removal was conducted 4 months later for both eyes. Intravitreal injections were conducted eight times separately for both eyes. VA of the patient was 20/20 in both eyes at the last follow-up.

### Patient 3

A 50-year-old male experienced blurred vision for both eyes 4 weeks after HAART initiation (3TC, EFV, and TDF). He had the herpes zoster infection three times since 2014. CD4 counts were 220/μl. VA was 20/25 in the right eye and 20/50 in the left eye. Ophthalmic examination showed retinal necrosis with vitritis (++) in both eyes in zones 2 and 3. He underwent PPV and silicone oil tamponades in both eyes and intravitreal injections six times in total. Vitreous DNA sequencing showed a CMV sequence of 60,527 copies. Silicone oil removal was conducted 4 months later. VA of the patient was 20/25 in the right eye and 20/40 in the left eye at the last follow-up.

### Patient 4

A 40-year-old male experienced blurred vision in the left eye 6 weeks after HAART initiation (3TC, EFV, and TDF). CD4 counts were 153/μl. VA was 20/66 in the left eye. His right eye has no light perception since his youth for an unknown reason. Ophthalmic examination showed retinal necrosis and hemorrhages with vitritis (++) in zones 1–3. He underwent PPV and intravitreal injection for the left eye. Afterward, immediate PPV and silicone oil tamponades were operated on due to vitreous hemorrhage for retinal holes 1 week after surgery. Moreover, regular intravitreal injections were performed six times in total. Vitreous DNA sequencing showed a CMV sequence of 206,679 copies. Silicone oil removal was conducted 6 months later. His VA of the left eye was 20/50 at the last follow-up.

## Discussion

In general, CMV retinitis was a severe opportunistic infection due to immunodeficiency with minimal intraocular inflammation, which has been extensively described in patients with HIV under ineffective treatment. We found that a small number of patients with CMV retinitis suffered retinitis progression and vitritis rapidly with HAART initiation. The results of this case series suggested that the pattern of CMVR of HIV patients might have changed by the introduction of HAART.

Traditionally, vitritis has not previously been associated with AIDS-related CMV retinitis. In 1997, Jacobson et al. firstly reported 5 cases of CMV retinitis with immune response 4–7 weeks after HAART (8). In their report, two patients had vitritis that was noted early in the course of the retinitis, and the vitritis occurred in patients who have a history of very low CD4 counts before HAART initiation. All 5 patients had recovered from the retinitis after a 7-month follow-up.

In 2014, Ruiz-Cruz et al. described a case series of patients with either new-onset CMV retinitis or CMV retinitis relapse (increasing border activity) soon after initiating HAART. They named it CMV-IRR, which could be described as a HAART-induced inflammatory immune response to subclinical CMV ocular infection (4). Most of them showed immune recovery, manifested as a rise of CD4 T cells and occurred within 2 months after HAART. Moreover, they suggested a hypothesis that active CMV retinitis might be an initial stage of a continual process leading to healed CMV retinitis observed in IRU.

However, some studies provided no evidence for the proposed “immune recovery retinitis.” The long-term result of CMVR in patients with HIV indicated that immune recovery with HAART can inactivate CMV retinitis lesions, even without anti-CMV drugs (9). The recent large-scale study by Gary et al. indicated that severe opacity was associated with lower CD4T-lymphocyte count and CMVR lesions were getting inactive after immune recovery with HAART (10). Tural et al. and MacDonald et al. demonstrated that some patients who responded to combined antiretroviral treatment with an increase in CD4 T-lymphocyte levels regained the ability to suppress CMV without specific anti-CMV therapy (11, 12).

Cytomegalovirus retinitis activity (indicated by the corresponding surface area) is accurately reflected by the presence and level of CMV DNA in aqueous humor and vitreous humor (13). In our study, among the thirty patients with CMV retinitis, four (13.3%) were progressed with active CMVR lesions with immune recovery within 8 weeks of HAART initiation. They all had a history of low CD4 T cells and a subsequent good immunological response, indicating the immune recovery retinitis caused by the immune response of CMV infection. Furthermore, they suffered from severe vitritis and a high copy of CMV in the vitreous body, which demonstrated that an effective HAART regimen might fail to inactive the CMV retinitis lesions.

Immune recovery uveitis emerges as a cause of visual morbidity in AIDS patients with pre-existing CMVR several months to years of initiation of HAART (14–17). Patients with IRU were reported in the literature to have no active retinitis or detectable CMV DNA in peripheral blood (14). It is associated with the presence of inactive CMV retinitis, combination antiretroviral therapy with protease inhibitors, and evidence of at least partial immune reconstitution suggested by increased CD4+ cell counts. The study by Robinson et al. showed that patients with IRU had a 10-fold increase in the mean CD4 T cell at the time of examination when compared with the mean CD4 T cell count at the time of diagnosis of CMV retinitis (18).

The reason for some patients showing CMV-IRR was still unknown. CMV-IRR may be caused by an immune response against persistent CMV antigen in the eye, while the immune response to CMV antigens varies a lot in patients (18). The possible mechanisms of CMV-IRR development could be summarized as follows: CMV retinitis causes a breakdown in the blood-ocular barrier and may allow migration of inflammatory cells into the eye. Patients with IRU might suffer from CMV retinitis or systemic CMV infection at a very low level of CD4 counts before HAART initiation. The good immunological response and increased CD4 counts with HAART treatment activated the immune response to CMV antigens. The inflammation could be activated by CMV antigens expressed on latently infected cells, near the areas of previously active CMV retinitis. Schrier et al. previously have shown that this may be related to the predisposition to CMV retinitis (6). Further studies are necessary to evaluate the incidence and pathogenesis of this newly described syndrome.

Treatment of CMV-IRR has faced rigorous challenge with limited experience and severe retina inflammation and high intraocular CMV copies. Regular treatment of CMV retinitis includes systemic and ocular ganciclovir (topical and intravitreal). Traditionally, weekly injections are advised until active CMV retinitis lesions are resolved (19). The concentration of intravitreal ganciclovir varies from 0.4 to 4 mg/0.1 ml in different research studies (20–24). A successful approach in South Africa gave biweekly injections of 2 mg of ganciclovir for the first 2 weeks followed by weekly injections until immune reconstitution, and the patient has received systematic anti-CMV treatment for at least 3 months (25). IRU with severe vitreous inflammation and/or CME typically was treated with periocular corticosteroids (triamcinolone acetonide 40 mg) or intravitreal corticosteroids without complications (26–28). Furthermore, some authors recommend restarting anti-CMV therapy to prevent CMV reactivation following corticosteroid treatment (3, 29). In our study, we combined intravitreal injection of ganciclovir with dexamethasone to suppress the high copies of CMV and severe vitritis and retinitis. Systemic corticosteroids and non-steroidal anti-inflammatory agents were not utilized because of relative immune compromise (30).

Ganciclovir is one of the optimal strategies in treating CMV retinitis. After diffusing into CMV-infected cells, ganciclovir is phosphorylated by the viral kinase UL97 and then further phosphorylated by cellular kinases to ganciclovir triphosphate, which inhibits the viral DNA polymerase UL54. To inhibit the replication of wild-type strains of CMV *in vitro*, the inhibitory concentration IC50 of Ganciclovir is 0.25–1.22 mg/l (31). Based on our experience and previous reports about CMV retinitis (22, 23), weekly intravitreal injection of 2 mg ganciclovir was effective and safe in most cases. The average intravitreal injections were 7.5 times (range from 6 to 10 times) in the four patients until retinal lesions were stable.

The presence of silicone oil in the vitreous cavity might have been a protective factor (26). It is fairly safe to assume that the ERM seen in our three patients was the result of vitritis inflammation (15). Recently, ERM formation has been recognized as an inflammatory complication of IRU by Studies of the Ocular Complications of AIDS and is being used as a diagnostic criterion in the definition of IRU (15, 26).

All four patients had immediate PPV with or without silicone oil tamponades due to severe retinal complications even under effective treatment. One patient with bilateral CMV-IRR had a retinal detachment in the non-PPV eye with routine intravitreal injections 1 month after the CMV-IRR diagnosis. Another patient suffered from vitreous hemorrhage because of retinal holes 1 week after PPV surgery (without silicone oil tamponades). They all had silicone oil removal 4–6 months after the surgery. Future studies with a larger sample size are needed to clarify the treatment and prognosis of the diseases. Regular ophthalmologic follow-up has been recommended at 3-month intervals for patients with HIV. Our patients had the incident of CMV-IRR within 2 months after HIV initiation. CMV-IRR was a newly diagnosed ocular complication with progressive retinitis and vitritis under effective HAART. Two of our patients developed vitreous hemorrhage or retinal detachment during treatment. Thus, we suggested a closer ophthalmologic examination especially for those patients with CD4 counts less than 50 cells/μl. Pupil dilation and fundus examination should be performed to avoid missing the peripheral lesions.

## Conclusions

In conclusion, patients with HIV are recommended to have a thorough ophthalmic examination before HAART. Vitreous DNA sequencing should be adopted in patients with possible CMV retinitis lesions. Intravitreal ganciclovir and corticosteroids are safe and effective in CMV-IRR patients. PPV with silicone oil tamponades may be essential to avoid the complications of retinal detachment and vitreous hemorrhage.

## Data Availability Statement

The original contributions presented in the study are included in the article/supplementary material, further inquiries can be directed to the corresponding author/s.

## Ethics Statement

The studies involving human participants were reviewed and approved by Huashan Hospital affiliated to Fudan University. The patients/participants provided their written informed consent to participate in this study. Written informed consent was obtained from the individual(s) for the publication of any potentially identifiable images or data included in this article.

## Author Contributions

Research design was conducted by ZW, YQ, and HL. Data collection was undertaken by LW, YQ, and JJ. Data analysis and interpretation were performed by XC, YQ, and LW. The manuscript was finished by YQ, LW, JJ, JS, HW, XC, HL, and ZW and revised by ZW and HL. All authors contributed to the article and approved the submitted version.

## Funding

This study was supported by the National Natural Science Foundation of China NSFC, no. 81900897 and Shanghai Science and Technology 20Y11910800. The sponsor or funding organization had no role in the design or conduct of this research.

## Conflict of Interest

The authors declare that the research was conducted in the absence of any commercial or financial relationships that could be construed as a potential conflict of interest.

## Publisher's Note

All claims expressed in this article are solely those of the authors and do not necessarily represent those of their affiliated organizations, or those of the publisher, the editors and the reviewers. Any product that may be evaluated in this article, or claim that may be made by its manufacturer, is not guaranteed or endorsed by the publisher.
